# NF-κB Signaling in Gastric Cancer

**DOI:** 10.3390/toxins9040119

**Published:** 2017-03-28

**Authors:** Olga Sokolova, Michael Naumann

**Affiliations:** Institute of Experimental Internal Medicine, Otto von Guericke University Magdeburg, Magdeburg 39120, Germany; Olga.Sokolova@med.ovgu.de

**Keywords:** NF-κB, inflammation, gastric cancer, *Helicobacter pylori*, VacA toxin, Type 4 secretion system, tumor microenvironment, chemoresistance

## Abstract

Gastric cancer is a leading cause of cancer death worldwide. Diet, obesity, smoking and chronic infections, especially with *Helicobacter pylori,* contribute to stomach cancer development. *H. pylori* possesses a variety of virulence factors including encoded factors from the cytotoxin-associated gene pathogenicity island (*cag*PAI) or vacuolating cytotoxin A (VacA). Most of the *cag*PAI-encoded products form a type 4 secretion system (T4SS), a pilus-like macromolecular transporter, which translocates CagA into the cytoplasm of the host cell. Only *H. pylori* strains carrying the *cag*PAI induce the transcription factor NF-κB, but CagA and VacA are dispensable for direct NF-κB activation. NF-κB-driven gene products include cytokines/chemokines, growth factors, anti-apoptotic factors, angiogenesis regulators and metalloproteinases. Many of the genes transcribed by NF-κB promote gastric carcinogenesis. Since it has been shown that chemotherapy-caused cellular stress could elicit activation of the survival factor NF-κB, which leads to acquisition of chemoresistance, the NF-κB system is recommended for therapeutic targeting. Research is motivated for further search of predisposing conditions, diagnostic markers and efficient drugs to improve significantly the overall survival of patients. In this review, we provide an overview about mechanisms and consequences of NF-κB activation in gastric mucosa in order to understand the role of NF-κB in gastric carcinogenesis.

## 1. Introduction

Gastric cancer is a high-recurrence-rate tumor, which is represented in about 90% cases by adenocarcinomas originated from the gastric mucosa glands, and can be further classified into intestinal (follows intestinal metaplasia and gastric atrophy) and diffuse (follows pan gastritis without atrophy) types. Anatomically, gastric cancer arises as cardia cancers and, in the majority of cases, as non-cardia tumors [1].

Gastric cancer remains the third leading cause of cancer death worldwide, following lung and liver cancers [2]. Nowadays, the highest stomach cancer incidence and mortality rate exist in Eastern Asia and Latin America [3]. In both low- and high-risk populations, dietary patterns, obesity, smoking and chronic infections, especially with *H. pylori*, contribute to stomach cancer development in addition to hereditary factors [4,5].

Estimation of the risk factors allowed useful cancer prevention strategies, which led to the declining occurrence of, e.g., *H. pylori*-associated non-cardia gastric cancer [4,6]. However, rates of gastric cardia cancer remain increased in Western countries [4], which motivates for further search of predisposing conditions and diagnostic markers. In case of advanced/relapsed gastric cancer, neither surgery nor chemotherapy improves significantly the overall survival of patients. Thus, clinical practice strongly needs efficient drugs, which are selective towards deregulated molecular networks in cancer cells [7,8].

Nuclear factor kappa B (NF-κB) refers to a group of transcription factors (RelA, RelB, c-Rel, NF-κB1/p50 and NF-κB2/p52), which form homo- and heterodimers and upregulate or suppress expression of many genes [9]. The NF-κB-driven gene products include cytokines/chemokines IL-1, IL-8, TNF, IL-6, MCP-1, pro- and anti-apoptotic factors cIAPs, c-FLIP, A20, Bcl-XL, angiogenesis regulator vascular endothelial growth factor (VEGF), matrix metalloproteinases (MMP)-2, MMP-9 in non-transformed or tumor cells in response to a variety of stimuli, including growth factors, cytokines, hormones, microbial and chemical compounds [10]. Many of the transcribed molecules promote gastric carcinogenesis; this recommends the NF-κB system for therapeutic targeting [11]. NF-κB activity is controlled through intracellular localization and protein modifications. NF-κB factors localize in the cytosol and are sequestered by IκB-molecules. In stimulatory conditions, IκBs undergo ubiquitinylation and degradation, which allow translocation of the NF-κB dimers into the nucleus [9].

In the early 2000-s, it became evident that the NF-κB system could be deregulated in gastric cancer [12,13], but the correlation between NF-κB activity and certain clinicopathological features is still not clear. It was reported that high nuclear abundance of RelA (by immunohistochemical analysis) in surgically resected specimens was related to tumor progression and predictable for a poor patient survival [14]. In contrary, high NF-κB activity in early-stage gastric carcinoma was found to correlate with better prognosis [15]. In human stage IV gastric carcinoma, RelA expression was found to decrease, and a group of patients with negative RelA demonstrated significantly increased overall survival after treatment with paclitaxel/LV5Fu2 or FOLFOX [16]. Later, a link between high cytoplasmic but not nuclear RelA and a worse survival of gastric cancer patients was described [17]. However, further research demonstrated that RelA and NF-κB1/p50 were up-regulated in gastric cancer and cancer cell lines [18]. Mean expression levels of phosphorylated RelA were higher in the diffuse type of gastric adenocarcinoma in comparison to intestinal type [19]. RelA expression in gastric cancer tissue strongly correlated with abundance of other tumor- and metastasis-promoting markers, including STAT3, MMP-9 [20], IL-6 and VEGF [21,22]. siRNA-mediated knockdown of RelA and NF-κB1/p50 had an anti-oncogenic effect both in vitro and in vivo [18].

It has been shown that chemotherapy itself, namely chemotherapy-caused cellular stress, can in some cases elicit NF-κB activation in gastric cancer cell lines, which supports cell survival leading to acquisition of chemoresistancy [23,24]. Nowadays, NF-κB signaling cascade accounts as one of important triggers in inflammation-induced cancerogenesis, including gastrointestinal malignancies [25,26]. In this review, we provide an overview about mechanisms and consequences of NF-κB activation in gastric mucosa in order to understand the role of NF-κB in gastric carcinogenesis.

## 2. Infections and NF-κB Regulation in Gastric Mucosa

Infections elicit inflammation, which is a complex innate and adaptive immune response aimed to remove pathogen and activate tissue repair. Insufficient elimination of the pathogen (chronic infection) leads to a sustained inflammatory environment, where cell populations involved are stimulated with locally produced pro-inflammatory mediators. This alters cellular homeodynamics and leads to accumulation of genetic and epigenetic changes in differentiated and stem cells. In the stomach, chronic inflammation is the initial step towards atrophy, metaplasia and dysplasia, and a potent promoter of cancer development. Finally, pathogen-induced inflammation becomes a cancer-induced inflammation, where damaged tumor cells and their products modulate the immune response [27,28].

Chronic infection with *H. pylori* accounts for about 90% of cases of non-cardia gastric cancer worldwide [4]. Twenty years ago, several research groups reported that a direct contact of *H. pylori* with transformed gastric epithelial cells induced fast (within 30 min) activation of NF-κB, nuclear translocation of p50/RelA and p50/p50 dimers, and strong accumulation of IL-8 mRNA [29,30]. Further, a major role of NF-κB and a contributory role of AP-1 transcription factor in regulation of *H. pylori*-induced cytokine expression was shown [31].

Importantly, activation of NF-κB was demonstrated in stomach epithelial cells of *H. pylori* gastritis patients, meaning that the gastritis-attributable neutrophil infiltration can be a consequence of *H. pylori*-induced NF-κB-dependent chemokine production in epithelial cells of gastric mucosa [29,32]. At this particular time, it was also sought after bacterial factors and molecular mechanisms of NF-κB activation by *H. pylori*. It became clear that, unlike LPS from other Gram-negative bacteria, *H. pylori* lipopolysaccharide (LPS) did not activate NF-κB [30]. However, *H. pylori* strains carrying 40 kb gene cluster named *cag* pathogenicity island (*cag*PAI) were more potent inducers of NF-κB activity and IL-8 expression in comparison to *cag*PAI-negative strains [33]. Some of the *cag*PAI genes, e.g., *cagE*, *cagL* and *cagI*, were identified as crucial for *H. pylori*-induced NF-κB [34,35].

Most of the *cag*PAI-encoded products form the bacterial type 4 secretion system (T4SS), a pilus-like macromolecular transporter, which translocates bacterial effector CagA from *H. pylori* into the cytoplasm of the host cell [36]. Taken in account that fast activation of NF-κB in gastric cell culture requires functional T4SS, two mechanisms have been proposed: (i) T4SS provides translocation of a bacterial effector into the cytosol; and (ii) T4SS binds to some plasma membrane receptors of the host cell and this launches signaling leading to NF-κB activation. CagA shows complex manipulation of host cell signaling [37], and ectopic expression of the *H. pylori* CagA protein in mice led to development of gastrointestinal carcinomas and hematopoietic malignancies [38]. Immunohistochemical analysis of *H. pylori*-positive metaplasia, dysplasia and carcinoma specimens demonstrated higher positive RelA rates in samples from CagA-positive patients (measured in sera by ELISA) in comparison to CagA-negative ones. Strong correlation between higher expression levels of RelA, c-Myc, cyclin D1, Bcl-XL in intestinal but not diffuse types of gastric carcinoma was also found [39]. For direct and fast NF-κB activation in gastric epithelial cell lines, the translocated CagA is dispensable [40,41,42]. In contrary, it has been described that a *H. pylori* strain which expresses the CagA EPIYA-C motif induces phosphorylation of IκBα [43].

*H. pylori* peptidoglycans are putatively translocated via the T4SS into host cells. It has been shown that peptidoglycan cytoplasmic receptor the nucleotide-binding oligomerization domain 1 (NOD1) triggers activation of the serine-threonine kinase RICK and TNF receptor–associated factor 3 (TRAF3) leading to activation of IFN regulatory factor 7 (IRF7), and subsequently to IFN-β production in mice [44]. Further, it has been recently shown that NOD1 can suppress NF-κB activity and expression of NF-κB-driven intestinal epithelial-specific transcription factor caudal-related homeobox 2 (Cdx2) (metaplasia contributing) in normal and neoplastic gastric epithelial cells through activation of TRAF3. NOD1-deficient infected mice had decreased expression of TRAF3 but increased expression of RelA and Cdx2, and developed intestinal metaplasia [45].

A number of studies addressed in detail the interaction of *H. pylori* components with host cell surface receptors. *H. pylori* possesses a variety of virulence factors for successful colonization of the human stomach mucosa. In addition to *cag*PAI, these include urease, outer membrane proteins of the *Hop-* and the *Hor-*gene families, vacuolating cytotoxin A (VacA), gamma-glutamyl transpeptidase (GGT), etc. [46]. The Hop family includes adhesins the blood group antigen binding adhesin (BabA), the sialic acid-binding adhesin (SabA), the adherence-associated lipoproteins A and B (AlpA and AlpB), HopZ, HopQ, outer inflammatory protein (OipA) and five Hop porins [47,48]. HopQ binds to the carcinoembryonic antigen-related cell adhesion molecules (CEACAMs) 1, 3, 5 and 6 on the host cell surface, which strongly supports *H. pylori* adherence and can potentiate thereby T4SS function [48,49,50]. OipA can activate NF-κB in gastric epithelial cells *cag*PAI-independently [51]. Further, there exists evidence that T4SS protein CagL binds to integrins on the host cell plasma membrane in order to support CagA translocation [36].

*H. pylori* toxin VacA, which induces apoptosis via damaging mitochondria, seems to be not required for NF-κB activation [35]. Bacterial GGT activated NF-κB, presumably through H_2_O_2_ generation and oxidative damage in primary gastric- and AGS cells [52]. GGT is considered as an important virulence factor involved in regulation of the host cell apoptosis. Other potential *H. pylori* molecules involved in regulation of NF-κB and cytokine expression in a T4SS-independent manner include the TNFα-inducing protein (Tipα), a secreted protein, which binds to the cell-surface before it enters the gastric cell [53,54,55].

Investigations of the molecular mechanism responsible for NF-κB activation in infected gastric cancer cell lines revealed the involvement of the IκB kinases (IKK) complex downstream of TRAF6-TAK1 signaling, similar to IL-1β-induced NF-κB regulation [56,57,58]. c-Src kinase binds transiently to IKKβ and phosphorylates IKKβ, and contributes at least in part to NF-κB activation in response to *H. pylori* infection [59]. Protein kinases Cα, δ, θ and the CARMA3-Bcl10-MALT1 complex are supposedly not involved in *H. pylori*-induced activation of NF-κB [60]. However, following long infection with *H. pylori*, many factors could be transcriptionally regulated with contributory roles in NF-κB activation [61]. Thus, despite obvious efforts, *H. pylori* factors and host cell membrane-associated mechanisms responsible for NF-κB activation in epithelial cells remain unresolved.

Another pathogen, *Mycoplasma hyorhinis* seems to be able to participate in gastric cancer progression. For example, *M. hyorhinis* membrane protein p37 forms a complex with the host epidermal growth factor (EGF) receptor and Annexin A2, and triggers thereby NF-κB activity required for cancer cells migration [62].

*Epstein-Barr virus* (*EBV*) has been suggested to promote carcinogenesis in gastric tissue. About 10% of gastric cancers, including lymphoepithelioma-like cancer, are positive for *EBV*, have defined clinicopathological features, locate in the proximal stomach and belong more frequently to diffuse or mixed types, in comparison to *EBV*-negative tumors [63,64]. Immunohistochemical analysis revealed that loss of the cell cycle regulators p21, p27, p16 and tumor suppressor APC, as well as increase of cyclin D1 and nuclear NF-κB accumulation were more often found in *EBV*-positive gastric carcinomas than in *EBV*-negative tumors [64]. In gastric cancer cell lines, expression of *EBV*-encoded latency I proteins BARF1 and Latent Membrane Protein-2A could trigger NF-κB activity and up-regulate thereby cyclin D1 and survivin, respectively, which rescued *EBV*-infected epithelial cells from some types of apoptosis and facilitated cancer progression [65,66].

Interestingly, *EBV* can produce miRNAs, some of which are highly expressed in *EBV*-associated gastric carcinomas and function to down-regulate expression of e.g., pro- and anti-apoptotic host proteins like Bid and Tax1-binding protein 1 (TAX1BP1), respectively [67,68]. In gastric cancer AGS cell line, viral miR-BART15-3p targets anti-apoptotic TAX1BP1 which promotes cell death and increases chemosensitivity, but simultaneously activates NF-κB, probably by suppressing A20, a TAX1BP1-dependent NF-κB negative regulator [68].

## 3. Pro-Inflammatory Mediators and Growth Factors in the Gastric Tumor Microenvironment

Cancerogenesis is a multistep process, which includes initiation, promotion and progression. In addition to initial DNA damage, a number of pro-inflammatory mediators, including TNF, IL-1, IL-6, stimulate phosphorylation of intracellular kinases, which induce transcription factors, e.g., NF-κB and AP-1, and early response genes. This effect is similar to the action of classical chemical tumor promoters TPA and okadaic acid, which support growth and proliferation of cells via activation of cell signal transduction [54].

In stomach, the pro-inflammatory mediators are secreted by cells of the gastric mucosa or carcinoma (autocrine route) or/and by infiltrating macrophages, neutrophils, lymphocytes (paracrine route) in response to pathogens (see above), some chemicals and physical insults [69,70,71]. High levels of IL-6, IL-10, IL-11, IL-32, and chemokine C-C motif ligands (CCL)7 and CCL21 in circulation, high expression of CXC chemokine receptor 4, chemokine C-C motif receptor 3 (CCR3), CCR4, CCR5, CCR7 and orphan nuclear receptor 4A2 in tumors were found to be associated with disease progression and an unfavorable prognosis in gastric cancer [72,73]. IL-6 and TNF are expressed at high levels in human gastric cancer samples. A positive correlation was found between the expression of IL-6 and NF-κB e.g., by immunohistochemical analysis. IL-6, NF-κB and VEGF protein and mRNA levels increased significantly in gastric cancer tissue compared with those in adjacent normal mucosa [21].

TNF can activate NF-κB-dependent CXCL1 and CXCL2 expression by stromal and endothelial cells. Further, these cytokines launch production of low-molecular weight calcium-binding proteins S100A8/9 in the stroma, immune and cancer cells [74]. S100A8/9 is able to activate NF-κB and mitogen-activated protein kinase (MAPK) p38 leading to MMP-2 and MMP-12 expression and increase thereby migration and invasion of gastric cancer cells [75]. Interestingly, TNF does not directly regulate calcium/calmodulin signaling in cancer cell culture [60]. Thus, TNF implicates other inflammatory mediators in the tumorigenic signaling network and facilitates tissue remodeling.

Already early investigations demonstrated the link between secretion of IL-1β at the site of gastric carcinoma tissue and activation of NF-κB in the tumor [76]. In support, infection of IL-1β-null mice with *H. pylori* did not activate NF-κB in the inflammatory and epithelial cells in gastric mucosa [70]. IL-1β-induced NF-κB-dependent target genes include, for example, retinoid x receptor α [77]; miR-425, which negatively regulates phosphatase and tensin homolog (PTEN) expression by targeting its 3′ UTR [78]; MMP-9 [79] in gastric cancer tissues and cell lines, that promotes cell proliferation and invasion. 

IL-17A is another inflammatory cytokine frequently detected in tumor microenvironment, which inversely correlates with five-year survival rates of gastric carcinoma patients [80]. Using gastric cancer cell lines, IL-17 has been shown to induce NF-κB and MAPKs leading to IL-8 secretion and to amplify thereby the inflammatory processes [80].

Some cytokines affect inflammation *via* inhibition of NF-κB in epithelial cells. IL-22, a member of the IL-10 family of cytokines, which is produced mainly by hematopoietic cells, binds its receptors expressed on the surface of epithelial cells, induces the Janus kinase (Jak)/STAT3 signaling and negatively regulates *H. pylori*-induced NF-κB-driven expression of CCL20 chemokine, which is aberrantly expressed in infected gastric mucosa [81]. This is one example of crosstalk between NF-κB and STAT3 regulatory pathways. Both transcription factors are stimulated by cytokines and cellular stress through different molecular mechanisms and regulate transcription (sometimes in a cooperative manner) of genes involved in anti-apoptotic, pro-proliferative and immune cellular responses. Thus, NF-κB and STAT3 contribute to gastric cancer development and progression [82,83]. In addition, NF-κB and STAT3 can synergistically influence the metastatic potential of gastric cancer cells through regulation of MMP-9 expression [20]. Inhibition of the constitutively active Jak/STAT3 in gastric tumor leads to reduced polymorphonuclear inflammation and inhibition of pro-inflammatory cytokines IL-11, IL-6 and IL-1β and reduction of tumor volume in mice [84].

One of the important NF-κB target genes is cyclooxygenase-2 (COX-2). COX-2 mediates production of thromboxane B2 and prostaglandins in gastric cancer cells, which promote cell proliferation and macrophage infiltration in stomach tissue [85]. Prostaglandins regulate NF-κB signaling by a positive feedback loop, which further amplifies and maintains inflammatory process and promotes formation of new blood vessels in tumor site [86,87].

Tumor-associated macrophages (TAMs) have immunosuppressive and tumor-promoting properties similar to the alternatively activated (M2) macrophages. Together with other subclasses of inflammatory cells and myeloid progenitors, they produce epithelial and stromal growth factors and matrix-remodeling enzymes [88], providing anti-apoptotic and pro-invasive signals. Both TAMs and gastric cancer cells express VEGF and VEGF-C in a NF-κB-dependent manner, which promotes angiogenesis and lymphangiogenesis of gastric cancer [22,89].

In addition, TAMs activate mesenchymal stem cells (MSCs), which further produce inflammatory cytokines. In cell culture experiments, the activated MSCs promoted both gastric epithelial cell and gastric cancer cell proliferation and migration. Active MSCs induce phosphorylation of NF-κB, ERK and STAT3, and inhibition of NF-κB activation by PDTC blocked the effects of activated MSCs on gastric cancer cells [90].

Cancer-associated fibroblasts (CAFs) is another type of cells involved in the paracrine secretion loops in gastric cancer. CAFs produce a variety of extracellular matrix components implicated in formation of the desmoplastic stroma [91]. CAFs isolated from gastric cancer patients secrete neuregulin1 (NRG1), which activate NF-κB in gastric cancer stem cells, potentiating their self-renewal. The overexpression of NRG1 in stromal cells and cancer cells was observed in tumor tissues of gastric cancer patients and was associated with clinical stage lymph node metastasis and survival in gastric cancer patients [92]. Both inflammation and cancer are metabolically costly processes, which require increased oxygen consumption. In conjunction with some vascular dysfunction, it predisposes tissue to hypoxia and further activation of hypoxia-inducible transcription factors (HIF)s. Cross-talk between NF-κB and HIFs attracts more attention nowadays [93,94].

Biglycan (BGN), an important component of the extracellular matrix, is overexpressed in gastric cancer tissues and promotes cancer metastasis. Stimulation of endothelial cells with BGN increased the interaction between NF-κB and the HIF-1α promoter, leading to enhanced promoter activity and to increased HIF-1α mRNA levels, as well as to augmented HIF-1 activity that resulted in VEGF expression [95].

Hepatocyte growth factor (HGF) was found to be expressed in human gastric cancer tissue [96]. HGF can be produced by CAFs [91]. In gastric cancer cells, HGF activates PI3K/AKT signaling leading to NF-κB activation and expression of Lipocalin 2 (a transporter of a small lipophilic ligand, which forms a heterodimer with MMP-9 in several cancer types), MMP-9 and heparanase (a sheddase of associated cytokines in several types of tumors), which finally promotes cell migration and invasion [96,97].

Cytokine bone morphogenetic protein (BMP)-2, a member of the transforming growth factor-β (TGF-β) superfamily, regulates cell proliferation, differentiation and organogenesis, e.g., promotes formation of bone and cartilage, and stomach glands. Increased serum levels of BMP-2 correlate with the grade of tumor histology and the depth of invasion in gastric cancer [98]. In gastric cancer cells, BMP-2 enhanced the phosphorylation/degradation of IκBα and the nuclear translocation/activation of NF-κB, as well as phosphorylation of AKT and ERK, leading to MMP-9 expression [99].

## 4. Tumor-Promoting Effectors and NF-κB Regulation in Gastric Tumors

A number of intracellular proteins with tumor-promoting or suppressing properties are aberrantly expressed during multistep gastric tumorigenesis and promote the pathology by regulating NF-κB activity. TLR4 is expressed in gastric cancer presumably in a stage-dependent manner [100]. TLR4 abundance could be regulated by MeCP2/HDAC1 repressor complex through epigenetic modification of DNA and histones on the TLR4 promoter and by Sp1, which activates TLR4 expression by hypomethylation and NF-κB signaling in gastric cancer cells [101]. TLR4 may participate in cancer-related inflammation via e.g., up-regulation of an oncogenic protein Astrocyte-elevated gene-1 (AEG-1) in gastric cancer tissue. AEG-1 binds to RelA and promotes its nuclear translocation, positively regulates IL-8 expression and cancer progression [102]. It has been demonstrated that stimulation of TLR4 with *Echerichia coli* LPS for 24 h in TLR4-positive gastric cancer cell lines, including BGC-823 and SGC-7901, induced production of mitochondrial ROS, AKT phosphorylation and RelA nuclear translocation, and enhanced proliferation without affecting apoptosis [100].

Calcium/calmodulin-dependent protein kinase II (CaMKII) phosphorylated at Thr286 is increased in metastatic gastric tissues in comparison to non-metastatic tissues. In a gastric cell line, overexpression of constitutively active H286R CaMKII enhanced NF-κB- and AKT-mediated MMP-9 production, which supported cellular migration and invasion [103]. Infection with *H. pylori* can contribute to this effect by triggering calmodulin [60]. 

Zipper-interacting Protein Kinase (ZIPK), a member of the death-associated protein kinase family, is expressed in human primary gastric cancer and the matched metastatic lymph node samples (by immunohistochemistry), and can be used as a prognostic marker. ZIPK promoted expression of β-catenin, Snail, Slug, and activated AKT and NF-κB leading to the epithelial-to-mesenchymal transition and metastasis in vitro and in nude mice [104].

Constitutively active PI3K or decreased phosphatase PTEN expression, as well as stimulation with EGF positively regulate Integrin-linked kinase (ILK). ILK facilitates the formation of the IQ motif-containing GTPase-activating protein 1 (IQGAP1)-Ras complex leading to activation of Ras/c-Raf/MEK1/2/ERK1/2, ribosomal S6 kinase and NF-κB signaling in AGS, SNU-1, MKN45, GES-1 gastric cancer cell lines. This signaling promoted cell growth, migration and reduced the sensitivity of gastric cancer cells to the anticancer agents 5-fluorouracil and cisplatin [105].

Osteopontin (OPN), a secretory extracellular matrix phosphoglycoprotein, which was originally identified in bone tissue, function to control biomineralization, osteoclast differentiation and bone resorption. In gastric cancer tissues, OPN is diffusely located in the cytoplasm of tumor cells and TAMs, which implicates interactions between cancer cells and tumor stroma. *H. pylori* oncoprotein CagA promotes OPN expression in gastric tumor cells [106]. OPN regulates proliferation, survival, adhesion, migration, invasion, angiogenesis and malfunction of TAMs, and degradation of extracellular matrix. Overexpression of OPN in gastric cancer correlates with poor overall survival and clinical features in patients [107]. OPN was proposed as a promising therapeutic target in gastric cancer. In gastric cancer cell lines, experimental silencing of OPN inhibited the MAPK, PI3K and NF-κB pathways, and OPN-mediated NF-κB activity was reduced in the presence of MAPK or PI3K inhibitors. OPN-directed NF-κB activation promotes the expression of MMP-2, MMP-9, urokinase-type plasminogen activator (uPA), the inhibition of caspase-3 [108].

High levels of phosphatase of regenerating liver-3 (PRL-3) contribute to gastric cancer progression and predict poor overall survival. PRL-3 induced phosphorylation of RelA, which promoted expression of HIF-1α and its transcriptional target miR-210, which was associated with increased invasiveness of cancer cells [109].

Angiogenesis-regulating resistin-like molecule-α (RELM-α) is overexpressed in gastric tumors and associates with advanced stage and tumor size [110]. The silencing of RELM-α expression by Ad5/F35-siRNA treatment significantly inhibited NF-κB activation and attenuated VEGF and MMP-9 expression as well as cell migratory and invasive ability in SGC7901 and MKN45 gastric cancer cells [111].

The expression of high mobility group box 1 (HMGB1), a novel inflammatory molecule, is increased in the gastric adenocarcinoma tissues compared with that in adjacent non-cancer tissues, and correlates with the metastasis. Knockdown of HMGB1 by shRNA downregulated the expression of RelA, PCNA and MMP-9 and inhibited proliferation and invasiveness of SGC-7901 and AGS cells. In SGC7901 subcutaneous nude mouse model, treatment with Lv-shHMGB1 led to reduction in tumor volumes [112].

Transmembrane protease serine 4 (TMPRSS4), a type-II transmembrane serine protease, is frequently upregulated in human cancers. Overexpression of TMPRSS4 in AGS and MKN-45 gastric cancer cells down-regulated the level of IκBα and induced nuclear NF-κB accumulation, enhanced expression and secretion of MMP-9, and promoted cell migration and invasion [113].

Deleted in breast cancer 1 (DBC1), a nuclear protein encoded by a gene on 8p21, is overexpressed in several human cancers, associates with TNM stage, lymph node metastasis and indicates for a poor prognosis. In vitro experiments showed that DBC1 expression promoted anoikis resistance in gastric cancer cells via activating the IKK-β/NF-κB signaling pathway, which may be beneficial for metastasis [114]. DBC1 has been proposed as a co-factor for IKKβ that stimulates its kinase activity, promoting RelA phosphorylation and transcriptional activity towards target genes such as *CFLAR* (c-FLIP) and *bcl-xl* in breast cancer [115].

Dopamine and cAMP-regulated phosphoprotein, Mr 32,000 (DARPP-32) is overexpressed during the gastric carcinogenesis and might inhibit TRAIL-induced caspase-dependent RelA cleavage [116].

## 5. Tumor Suppressors and NF-κB Regulation in Gastric Cancer

Several molecules were described to function as tumor suppressors in gastric cancer through inhibiting NF-κB signaling. These include trefoil factor (TFF) 1, Inhibitor of growth 4 (ING4), metallothionein 2A (MT2A), dapper homolog 1 (DACT1), zinc finger transcription factor ZNF382. Among the 3 TFFs, TFF1 is produced mainly by the gastric epithelia, especially by the mucus-secreting pit cells of the corpus, antrum and pylorus. TFF1 supports cellular differentiation and participates in maintenance of the integrity of the mucus layer. Expression of TFF1 is induced by mucosal injury, and the loss of this small protein accompanies about 2/3 of gastric carcinomas [117,118]. Markedly, *Tff1*-/- knockout mice exhibit inflammation in the gastric mucosa as well as “age-dependent carcinogenic histological changes in the pyloric antrum of the gastric mucosa, progressing from gastritis to hyperplasia, low-grade dysplasia, high-grade dysplasia, and ultimately malignant adenocarcinoma” [117]. Molecular studies demonstrated that loss of TFF1 correlates with increased levels of TNF-activated NF-κB and overexpression of Aurora Kinase A (AURKA). AURKA, a mitosis-regulating serine-threonine kinase, has been found to regulate NF-κB activity by direct binding and phosphorylating IκBα [119]. The reconstitution of TFF1 expression in gastric cancer cells suppressed *H. pylori* mediated NF-κB activation and expression of cytokine genes (TNF, IL-1β, CCL5, and IL-4 receptor) [120]. A small selective inhibitor of AURKA MLN8237 (Alisertib) suppressed NF-κB activity in human gastric cancer samples and mouse epithelial cells, and reduced expression of NF-κB target genes that regulate inflammation and cell survival. Inhibition of AURKA also reduced growth of xenograft tumors from human gastric cancer cells in mice and reversed the development of gastric tumors in *Tff1*-/- knockout mice [119].

ING4 mRNAs and protein in human gastric carcinoma tissues and cells were significantly lower compared to cells from normal tissue. Overexpression of ING4 resulted in the down-regulation of RelA, p-IκBα, MMP-9 and uPA proteins and inhibited proliferation and invasion in gastric carcinoma cell lines MKN-28, SGC-7901 and MKN-45 [121].

Stress protein MT2A plays a protective role in gastric mucosal barrier. Decreased MT2A expression was detected in cell lines and primary tumors of gastric cancer, and was associated with poor prognosis, advanced TNM stage, and down-regulation of IκBα expression. MT2A inhibited cell growth through apoptosis and G2/M arrest, which was accompanied by a decrease in NF-κB activity and cyclin D1 expression [122].

Dapper homolog 1 (DACT1), a disheveled partner in the planar cell polarity pathway, and a transcription regulator zink finger ZNF382 were found to be frequently silenced in gastric cancer cell lines and in primary gastric cancers via promoter hypermethylation. DACT1 methylation was associated with tumor metastasis, invasion and advanced stage. Overexpression of DACT1 or ZNF382 in silenced gastric cancer cell lines suppressed colony formation, proliferation and induced cell apoptosis. DACT1 inhibited NF-κB activation and downstream expression of Bcl-2, Bcl-XL, IL-8 and TNF [123]. ZNF382 inhibited NF-κB and AP-1 signaling and the expression of oncogenes MYC, MITF, HMGA2, and CDK6 as well as the NF-κB upstream factors STAT3, STAT5B and ID1 most likely through interaction and silencing of heterochromatin [124].

The carboxyl terminus of Hsc-70-interacting protein (CHIP), a U-box-type ubiquitin ligase, was found to be frequently decreased in gastric cancer tissues, indicating for an unfavorable prognosis [123]. CHIP overexpression in the AGS gastric cancer cells inhibited RelA and RelB via most likely TRAF2 reduction. This impaired tumor growth, the migration and invasion of the cells, and MMP-2, MMP-9, integrin β1 and Bcl-2 were involved in these processes [125].

Gastrokines play a role in maintaining the gastric mucosal integrity and function, and exhibit chemoattractant properties for the migration of lymphocytes [126,127]. GKN1 is a stomach-specific protein that is expressed in gastric mucosa, but not in primary tumors or cancer cell lines. Xing et al. described a gradual decrease in GKN1 expression from normal mucosa to dysplastic gastric tissue to gastric cancer, and demonstrated that low GKN1 expression was associated with metastasis [128]. GKN1-transfected AGS cells exhibited an inactivation and reduced expression of NF-κB and COX-2, induced production of inflammatory cytokines IL-8 and -17A, but decreased expression of IL-6 and -10 [126]. In addition, GNK1-dependent inactivation of NF-κB leads to diminished MMP-2 expression and mitigated invasion [128].

Similar to GKN1, insulin-like growth factor binding protein-3 (IGFBP-3) is a potent tumor suppressor, which is down-regulated in gastric cancer. Overexpression of IGFBP-3 resulted in significant inhibition of total and phosphorylated RelA and IκB-proteins in gastric cancer cells and in reduced expression of NF-κB-regulated cell adhesion molecules, ICAM-1 and VCAM-1 [129].

Long and small non-coding RNAs regulate gene transcription and transcripts processing. Over the past decade, emerging evidence has revealed an involvement of non-coding RNAs in gastric tumorigenesis. In human gastric carcinoma, miR-7 and miR-9, which target RelA, c-Fos and NF-κB1/p50, respectively, and miR-508-3p, which targets both RelA and NF-κB1/p50, were down-regulated. In vitro, overexpression of the miRs repressed expression of their targets and prevented gastric cancer cell proliferation [18,130,131]. In addition, NF-κB has been shown to repress miR-7 transcription, and decrease in miR-7 expression can be caused by aberrant NF-κB activation following *H. pylori* infection [130]. Long non-coding RNA BANCR, which high expression correlates with proliferation and migration of gastric cancer cells, can positively regulate NF-κB via inhibiting miR9 [132].

MiR-218 directly targets epidermal growth factor receptor-co-amplified and overexpressed protein (ECOP), which regulates the NF-κB transcriptional activity and is associated with apoptotic response [133]. MiR-128b directly targets PDK1, which may regulate AKT/NF-κB signaling [134], and overexpression of miR-128b or -218 suppressed cell proliferation and increased apoptosis in vitro [133,134].

Similarly, miR-500 is upregulated in human gastric cancer, which correlates with malignant progression and poor survival. In gastric cancer cell lines, miR-500 directly repressed CYLD, OTUD7B, and the A20 complex component TAX1BP1 which led to sustained NF-κB activation [135]. Similar effect has miR-362, which targets CYLD [136].

miR-146a is up-regulated in a mouse model of gastric cancer and in human gastric adenocarcinomas, where it targets caspase recruitment domain-containing protein 10 (CARD10) and COP9 signalosome (CSN) complex subunit CSN8 [137]. Via this mechanism, miR-146a suppresses GPCR-mediated activation of NF-κB and expression of tumor-promoting cytokines and growth factors. Interestingly, miR-146a is NF-κB-dependently regulated in monocytes, which is promoted in response to a variety of microbial components and pro-inflammatory cytokines, and targets TRAF6 and IL-1 receptor-associated kinase 1 (IRAK1) [138]. Therefore, miR-146 seems to control NF-κB activation by a negative feedback mechanism. MiR-372 can regulate NF-κB via suppression of TNF-induced protein 1 (TNFAIP1) [139], and miR-1228* regulates NF-κB via Casein kinase CK2A2 [140].

MiR-155-5p targets RelA and IKKε. Its downregulation induces bone marrow-MSC to acquire a gastric cancer tissue-MSC-like phenotype, a classic phenotype of reactive stroma cells with stronger gastric cancer promoting capacity. This suggests a novel mechanism underlying the cancer associated MSC remodeling in the tumor microenvironment and offers an effective target and approach for gastric cancer therapy [141].

## 6. Polymorphisms in NF-κB Genes in Gastric Carcinoma

Genetic polymorphisms have been suggested as a risk factor for precancerous lesions and gastric cancer development. A functional polymorphism of the *NF*κ*B1/p50* promoter was first identified by [142]. They demonstrated that the *NF*κ*B1/p50* promoter containing the -94delATTG allele was less active than that containing the -94insATTG allele, and was associated with susceptibility to ulcerative colitis. Lo et al. [143] found that the insertion allele-type, genotypes with ins/ins, as well as ins allele carrier (ins/ins + ins/del) were more frequent in gastric cancer patients, especially in patients 65 years old, but not in younger patients. The authors suggested that the functional *NF*κ*B1/p50* polymorphism might moderately increase the pathology risk [143]. Of note, the polymorphism did not correlate with clinicopathologic factors and patient survival [143,144].

Recently, Arisawa et al. [145] demonstrated that the rs28362941 del/del and the rs78696119 GG homozygotes were associated with the diffuse type of gastric cancer, especially in young subjects. In addition, both *NF*κ*B1/p50* promoter variants were associated with tumor progression such as tumor invasion and lymph node metastasis, in comparison to other genotypes.

Single-nucleotide polymorphisms (SNPs) within *NF*κ*B1/p50* introns were also reported. The patients with the A allele of the *NF*κ*B1/p50* rs230510 had significantly longer overall survival compared with those with the T/T genotype in both the Japanese and US cohort, indicating that the *NF*κ*B1/p50* intron-located rs230510 may be a promising prognostic marker in gastric cancer [146]. Homozygous rs4648068 GG was associated with an increased risk of gastric cancer, especially for the lymph node status and serosa invasion in Han Chinese population [147]. In addition, intensive staining for NF-κB1/p50 expression was observed in tissues of GG genotype patients, compared with those of GA group and AA genotype patients [148]. In vitro, the luciferase activity of homozygote-overexpressing group (pGL3-GG) was greater than that of the control (pGL3-AA), especially at the stimulation of LPS [148]. Thus, genotypes of NF-κB genes could be suitable as predictive biomarkers. 

## 7. NF-κB and Gastric Cancer Therapy

The upregulated NF-κB pathway and Th1-type cytokine response in gastric mucosa, e.g., in the individuals infected with *H. pylori*, alter the overall integrity of the gut epithelial barrier. Besides cytokines and their receptors, NF-κB induces transcription of extracellular matrix remodeling enzymes. Indirectly, via regulation of other transcription factors, NF-κB influences expression of VEGF and E-cadherin in different types of gastric carcinoma [149,150], promoting epithelial-to-mesenchymal transition, tumor angiogenesis and metastatic dissemination. A number of NF-κB-regulated gene products, including cyclin D1, COX-2, etc. have been closely linked with the development of chemoresistance in gastric cancer [151]. Thus, a control of NF-κB activity might be a useful therapeutic strategy to augment conventional therapy and circumvent chemoresistance in gastric cancer patients.

The gastric cancer is often diagnosed at an advanced stage, therefore conventional therapeutic approaches on the basis of tumor resection, radiation and chemotherapy do not considerably improve survival of gastric cancer patients. Recent insights into the molecular pathways involved in gastric carcinogenesis allowed creation of novel treatment options for gastric cancer patients. Trastuzumab, a HER-2-targeting antibody, was shown to improve survival of advanced gastric cancer patients with HER-2 overexpression in their tumor cells. Ramucirumab, a VEGF receptor-2-targeting antibody, is an approved drug in combination with capecitabine/fluorouracil (5-FU) and cisplatin chemotherapy [152], and other molecules against c-Met/HGF, mTOR, and EGF receptor are currently under investigation [7,153,154]. Interestingly, HER-2, VEGF receptor, c-Met, PI3K/AKT, mTOR, and EGF receptor have been shown to be regulated in *H. pylori* infection [155,156,157,158,159], and pro-apoptotic effects of HER-2- and PI3K/AKT-targeting drugs could be mediated by NF-κB inhibition [160,161].

NF-κB and integral components as suitable therapeutic targets have been also studied. Gastric mucosal epithelium of IKKβ-knockout mice exhibits fewer gastrointestinal tumors, less IL-1α secretion and more apoptotic cell death in response to N-methyl-N-nitrosourea (MNU) [162]. The IKKβ deficiency in mice gastric epithelial cells led to increased apoptosis, reactive oxygen species, and cellular necrosis, and resulted in down-regulation of anti-apoptotic genes in *H. felis* infection [163].

Inhibition of NF-κB by dehydroxymethylepoxyquinomicin (DHMEQ) led to diminished expression of integrins α1, α3 and β1 and adhesion of human gastric cancer cells NUGC-4 and 44As3Luc to the extracellular matrix. In mice, the viability of DHMEQ-pre-treated cells injected in the peritoneum was affected [164]. BAY11-7082 and BAY11-7085 inhibit IκBα phosphorylation, degradation and prevent nuclear NF-κB translocation. In gastric cancer cell line HGC-27, BAY11-7082 suppressed cyclin A and CDK-2 expression, induced S phase arrest and exhibited rapid and potent apoptotic effects, reproducible in xenografts [165].

Several drugs (paclitaxel, docetaxel, cisplatin, 5-FU, etc.) routinely used for gastric cancer treatment induce genotoxic stress but activate NF-κB leading to cell survival [24,166,167,168,169]. In these cases, the control of NF-κB could increase efficiency of the drug therapy. Indeed, intraperitoneal administrations of RelA siRNA or nafamostat mesilate (FUT-175), a synthetic serine protease inhibitor and an NF-κB inhibitor, in addition to paclitaxel were effective for suppressing peritoneal metastasis and increased survival time of mice with gastric cancer [170,171].

Inhibition of NF-κB with a sesquiterpene lactone parthenolide, inhibited cell growth in gastric cancer cell lines MKN-28, MKN-45 and MKN-74. In mice, parthenolide alone or synergistically with paclitaxel suppressed the disseminated peritoneal nodules. Moreover, the combined therapy of parthenolide and paclitaxel increased the survival time [172]. A protein-bound polysaccharide (PSK), which is used as a chemo-immunotherapy agent in the treatment of cancer in Asia, enhanced docetaxel-mediated tumor suppression in gastric cancer cells and in xenografts via suppression of NF-κB [166].

Simvastatin, a cholesterol-lowering drug, can sensitize the gastric cancer to the antitumor effects of capecitabine through suppressing the constitutive activation of NF-κB and expression of COX-2, cyclin D1, Bcl-2, survivin, CXC motif receptor 4, and MMP-9 proteins in gastric cancer cells. In mice xenografts, simvastatin enhanced the apoptotic effects of capecitabine through suppression of NF-κB-regulated markers of proliferation, invasion, angiogenesis, and metastasis [151].

Many molecules involved in NF-κB signaling are regulated through ubiquitinylation. K48-linked ubiquitinylation creates a signal for proteasomal degradation of the labeled protein. 26S proteasome-dependent degradation of IκBs permits nuclear translocation of NF-κB. Inhibition of the proteasome by inhibitors, e.g., lactacystin and MG132, suppressed NF-κB activity and increased caspase-3 amount in gastric cancer cells [173]. Bortezomib, a selective inhibitor of the proteasome, exhibited apoptotic and anti-proliferative effects, resulting in a reduction in survival rates of gastric cancer cells in vitro and in subcutaneously transplanted nude mice. The induction of apoptosis was dependent on the inhibition of NF-κB activation, subsequent ROS production and JNK activation [174]. A phase II trial in patients with advanced gastric adenocarcinoma suggested bortezomib usage in combination with inhibitors of other oncogenic pathways [175].

A number of natural-anti-inflammatory agents, including resveratrol, curcumin and their synthetic analogues, have been proposed inhibit NF-κB and exert anti-inflammatory and anti-tumor properties [176,177,178,179]. Hormones and vitamins have low systemic toxicity and can enhance effects of anti-cancer drugs via diminishing NF-κB activity. Melatonin, synthesized by the pineal gland and released into the blood, appears to have antitumor properties. Melatonin inhibited nuclear translocation of NF-κB, activated the caspase-dependent apoptotic pathway via MAPKs and enhanced the anti-tumor effects of cisplatin in AGS cells [180]. Treatment of human gastric carcinoma SGC-7901 cells with vitamin E succinate (RRR-α-tocopheryl succinate) led to a decreased nuclear and cytosolic RelA protein amount, diminished NF-κB-DNA binding, decrease in protein expression of Bcl-2, increased protein levels of Bax, and cleaved caspase-9, caspase-3 and PARP. Pretreatment with NF-κB inhibitor dithiocarbamate reinforced all the above RRR-α-tocopheryl succinate-induced effects [181]. A vitamin E analogue γ-tocotrienol, which is already in clinical trials, can reduce the resistance and can potentiate the apoptotic effects of capecitabine against gastric cancer via inhibition of NF-κB and the NF-κB-regulated expression of COX-2, cyclin D1, and MMP-9 in various gastric cancer cell lines and in xenografts from human gastric cancer [182].

Several reports pointed also an NF-κB-inhibitory effect of probiotic bacteria (e.g., *Lactobacillus* species) or their extracts in gastric cancer cell lines [183,184].

Non-steroidal anti-inflammatory drugs (NSAIDs) or selective COX-2 inhibitors possess anti-metastatic activity, especially in case of the gastrointestinal tract cancers [185]. Besides inhibiting the conversion of arachidonic acid to prostaglandins, NSAIDs and COX-2 inhibitors can directly affect NF-κB activity. COX-2 inhibitor SC236 directly suppressed nuclear translocation of RelA [186]. Sulindac and its metabolites inhibited the NF-κB-mediated survival signals through inhibition of IKKβ by their direct interaction and enhanced TNF-induced apoptosis in human gastric MKN45 and cervical carcinoma HeLa cell lines [187]. NO-acetylsalicylic acid (ASA) and NO-naproxen reduced NF-κB protein levels, activated caspase-3 and inhibited HT-29 human colon cancer cell growth. In rats, treatments with NO-ASA, NONO-ASA, and NO-naproxen led to S-nitrosylation of RelA sulfhydryl residues in the stomach tissue, increases in plasma TNF, and reductions in mucosal PGE2 levels [188]. Thus, NSAIDs might be used for cancer prevention or augment the efficacy of more conventional therapeutic approaches in developed cancer.

Anti-microbial therapy is an important preventive strategy in gastric cancer. *H. pylori* eradication leads to a regression of atrophic gastritis and is often prescribed to the patients with premalignant gastric lesions [189]. What happens with NF-κB activity after *H. pylori* eradication in vivo has not been investigated. Proton pump inhibitors, which are used in complex therapy, can supposedly down-regulate NF-κB in cells [190].

Novel immunotherapy strategies are expected to target immune cells, their molecular mediators and effectors including NF-κB. Immunotherapy evaluated in gastrointestinal malignancies has been described recently [154,191,192].

## 8. Conclusions

**Figure 1:**
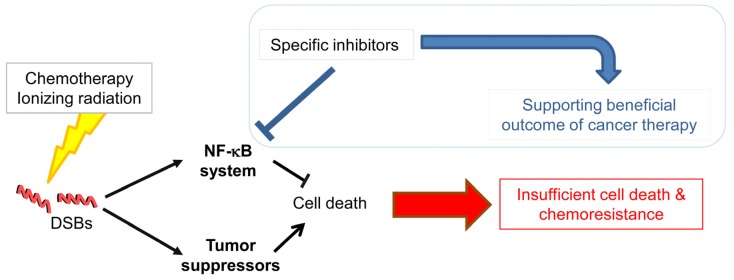
Beneficial outcome of cancer therapy by targeting the NF-κB system. Environmental stress and genetic predisposition could initiate gastric cancer development. Chemo/radiotherapy induces DNA double strand breaks (DSB) leading to the activation of the NF-κB system and tumor suppressors. Due to anti-apoptotic activity, NF-κB accounts for insufficient cell death and chemoresistance, thus, therapeutic targeting of factors, which promote the activation of NF-κB could support beneficial outcome of cancer therapy.

It is obvious that the NF-κB system is elementary in linking inflammation and cancer involving different cell populations of the stomach. The recruitment and activation of inflammatory cells trigger tumor progression and metastasis. NF-κB controls a variety of cellular processes regulating proliferation, evasion of apoptosis and angiogenesis. Therefore, the NF-κB system provides promising biomarkers for diagnostics and therapeutic targeting in cancer patients. Experimental and clinical data demonstrated that pharmacological inhibition of NF-κB could exert beneficial effects regarding the outcome of various anti-cancer therapies by promoting cell death (Figure 1). Dysregulation of cancer cells comprises a number of genetic alterations, which impacts on the signaling network and the control of cellular processes. Thus, therapeutic targeting of NF-κB could also elicit opposing effects. Therefore, in addition to intensive research of the NF-κB system and the identification of inhibiting compounds, further investigations are requested to understand the NF-κB molecular network for the development of synergistic complex therapies in gastric cancer.
